# The Efficiency of Platelet-Rich Plasma (PRP) in Treating Post-Burn and Surgical Scars: A Meta-Analysis Study

**DOI:** 10.3390/jcm14238490

**Published:** 2025-11-30

**Authors:** Ziyad Alharbi, Tala Zafar

**Affiliations:** 1 Plastic Surgery and Burn Unit, Dr. Soliman Fakeeh Hospital, Jeddah 23323, Saudi Arabia; zialharbi@fakeeh.care; 2 Department of Clinical Sciences, Fakeeh College for Medical Sciences, Jeddah 23323, Saudi Arabia; 3 College of Medicine, King Abdulaziz University (KAU), Jeddah 21589, Saudi Arabia

**Keywords:** burns, keloids, platelet rich plasma, PRP, post-surgical

## Abstract

**Background/Objectives:** Scarring frequently lowers quality of life and can have both functional and aesthetic impacts. Recently it has been debated whether platelet-rich plasma (PRP) can be used as a therapy modality for improving scar outcomes, given its abundance of cytokines and growth factors. This study attempts to methodically assess PRP’s efficacy in healing various scar types, such as burns, keloids, and postoperative scars, using randomized controlled trials (RCTs). **Methods:** A systematic search with PubMed, Google Scholar, Ovid Medline, Web of Science, and the Cochrane Central Register of Controlled Trials (CENTRAL) was conducted for RCTs, up to 12 October 2024, evaluating the use of PRP in scar treatment. Outcomes were assessed using validated tools, including the Patient and Observer Scar Assessment Scale (POSAS) and the Vancouver Scar Scale (VSS), with only one study using ultrasound imaging. The Cochrane Risk of Bias 2.0 tool was used to analyze potential bias. Standardized mean differences were computed for continuous outcomes, and meta-analyses were conducted with random-effects models. **Results:** A total of 11 RCTs involving 539 participants were included, covering 5 postoperative scars, 4 burn scars, and 2 keloid studies. Although several individual trials indicated enhancements with PRP, the pooled data revealed no statistically significant alterations in scar appearance at three months, as assessed by the Observer Scar Assessment Scale (OSAS) and VSS scores. A statistically significant enhancement in patient-reported outcomes utilizing the Patient Scar Assessment Scale (PSAS) was noted at 6 months (mean difference = −0.825; 95% CI: −1.561 to −0.090; *p* = 0.028), suggesting PRP’s efficacy in alleviating symptoms, including pain, itching, and stiffness. High heterogeneity was noted in several analyses, likely due to variability in PRP preparation methods, administration protocols, and scar types. **Conclusions:** This meta-analysis indicates that PRP may provide significant subjective enhancements in scar quality, particularly from the patient’s viewpoint. The results show the need for standardized PRP protocols, prolonged follow-up periods, and the integration of both patient-centered and objective outcome measures. PRP has significant potential in early postoperative wound healing; nevertheless, additional high-quality, long-term studies are required to clarify its role in mature or fibrotic scars.

## 1. Introduction

Burns are injuries to the skin, mucous membranes, and deep tissues, and in severe situations, they can injure internal organs and lead to death. Heat causes the majority of burn injuries; however, frostbites, chemical corrosives, electrical currents, and radiation are other sources of burns. Different sources can lead to different results; some may cause slight inflammation, while others result in ischemia, necrosis, shock, and death [1]. The severity of damage is classified according to its depth and size [2]. Burn injuries are extremely frequent, rating as the fourth most common traumatic incident worldwide. According to the WHO, 11 million cases from all different causes of burns occur annually worldwide, with a yearly average of 180,000 cases ending in death. Non-fatal cases are a leading cause of disability-adjusted life-years (DALYs), mainly in low and middle-income countries [3]. Superficial and partial superficial burns usually heal rapidly; however, deeper burns require more extensive care, as they have a higher risk of being infected and developing hyperplastic scars, which can lead to contracture deformities [4]. Scars develop as a natural part of the wound healing process. The final appearance of a scar is influenced by various factors, including an individual’s genetic makeup, the nature and severity of the injury, and the location of the scar. When skin is injured, the healing process begins with hemostasis, followed by inflammation, proliferation, and remodeling of the damaged tissue [5]. A keloid scar is a type of benign fibro-proliferative dermal growth that extends beyond the original wound site and infiltrates surrounding tissue as a consequence of abnormal wound healing. This occurs due to elevated levels of cytokines and growth factors, which leads to excessive production of the extracellular matrix, particularly collagen. Scars can pose significant cosmetic issues, and when paired with functional difficulties, like contractures, and symptoms, such as pruritus and pain, it can greatly impact a patient’s overall quality of life, physical status, and psychological health [6]. Various treatment options are suggested for keloids, and standard approaches can vary geographically. These include surgical removal, cryotherapy, laser therapy, radiofrequency treatment, and intralesional injections of steroids [7].

**Figure 1:**
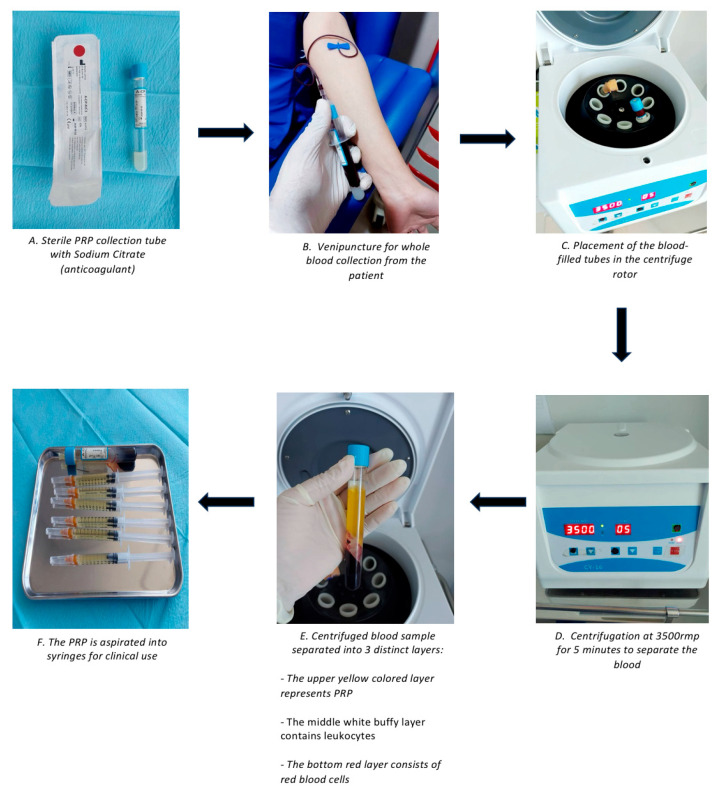
Overall PRP preparation process.

Platelet-rich plasma (PRP) is autologous blood that contains three times the number of platelets compared to normal blood. The molecular makeup of the plasma portion of PRP closely resembles that of blood. The latest studies have shown notable concentrations of dissolved proteins, including serum growth factors, albumins, globulins, fibrinogen, and several clotting factors that are crucial for the coagulation process [8]. Platelet-derived growth factor (PDGF) is released shortly after injury and stimulates the proliferation of fibroblast and smooth muscle cells, as well as the synthesis of collagen and angiogenesis. Vascular endothelial growth factor (VEGF) is a critical factor in angiogenesis, which is the formation new blood vessels in granulation tissue. Epidermal growth factor (EGF) helps to re-epithelialize and reduce scaring by promoting keratinocyte migration and proliferation. Angiogenesis, collagen deposition, and the formation of granulation tissue are all facilitated by fibroblast growth factor (FGF). Insulin-like growth factor (IGF-1), active in inflammatory and proliferative phases, enhances tissue repair and synergizes with PDGF and EGF. Fibroblast activity and ECM remodeling are regulated by transforming growth factor-beta (TGF-β) isoforms. TGF-β1 and β2 are associated with fibrosis, whereas TGF-β3 aids in the reduction of scar formation. Hepatocyte growth factor (HGF) and keratinocyte growth factor (KGF) promote epithelial healing, angiogenesis, and keratinocyte proliferation, resulting in faster wound closure. Together, these characteristics explain PRP’s regenerative potential in improving scar outcomes [9]. Platelet delta granules contain substances such as serotonin, histamine, dopamine, calcium, and adenosine, which work together with the growth factors mentioned earlier to facilitate membrane permeability and modulate inflammatory processes, helping in the process of wound healing. PRP is obtained by centrifuging whole blood, resulting in the separation of the blood into three distinct layers: platelet-poor plasma, platelet-rich plasma, and red blood cells (Figure 1) [10]. PRP can be classified into four types based on the concentration of platelets and leukocytes present in the plasma, as well as the fibrin content: pure platelet-rich plasma (P-PRP), leucocyte- and platelet-rich plasma (L-PRP), pure platelet-rich fibrin (P-PRF), and leucocyte- and platelet-rich fibrin (L-PRF) [1]. Both animal and human studies in fields such as oral and maxillofacial surgery, otolaryngology, plastic surgery, and general surgery suggest that PRP products may enhance the healing of soft tissues [11].

Despite the potential of PRP in burn wound treatment, there are limited reports on its application, and the clinical effectiveness of PRP for burns is still uncertain [4]. Clinicians are increasingly using PRP in keloid treatment, though these methods are largely based on empirical evidence and do not guarantee a cure. Nonetheless, efforts to find new scar treatment options are ongoing [7].

To the best of our knowledge, there is no prior single meta-analysis evaluating the effectiveness of PRP usage in burns and scars. The aim of this study was to conduct an extensive review of the existing literature and carry out a meta-analysis of RCTs evaluating the overall effectiveness of PRP in improving scar appearance and quality, including post-surgical scars, burns, and keloids, based on both clinical judgment and patient-reported satisfaction and in comparison to other treatments or no treatment.

## 2. Materials and Methods

### 2.1. Search Strategy

A systematic review was designed in accordance with the Preferred Reporting Items for Systematic Review and Meta-Analyses (PRISMA) guidelines [12]. A systemic literature review was conducted by the two authors from 1 to 12 October 2024 utilizing the following electronic databases: PubMed, Google Scholar, Ovid Medline, Web of Science, and Cochrane Central Register of Controlled Trials (CENTRAL). The search keywords included (effectiveness OR success OR safety OR satisfaction) AND (platelet rich plasma OR PRP OR platelet gel OR plasma gel) AND (scars OR hypertrophic scars OR surgical scars OR burns OR keloids). We examined articles based on established quality standards and reviewed the references of eligible studies. Ethical approval was not required for this study and registration had not been done.

### 2.2. Inclusion and Exclusion Criteria

In this systematic review, studies were selected based on predefined inclusion and exclusion criteria. The inclusion criteria were determined using the Population, Intervention, Comparison, Outcome, and Study design approach. Only RCT studies enrolling participants with keloids, hypertrophic, burn, or surgical scars were included. These studies used platelet-rich plasma in the form of injections or gels, leading to scar improvement outcomes assessed using the Vancouver Scar Scale (VSS), Visual Analog Scale (VAS), Patient Observer Scar Assessment Scale (POSAS), REEDA Scale, or ultrasound and photographs.

The exclusion criteria included published studies that were not RCTs, animal studies, studies written in languages other than English, studies focused on skin and non-dermatological disorders other than scars, studies focused exclusively on acne scars, studies focused on PRP composition, studies not focused on PRP treatment, studies that used PRP in combination therapy, and studies that did not on assess relevant outcomes.

### 2.3. Study Selection

The final compatible studies were obtained in a two-step process, with both steps carried out by the two authors. The initial step involved screening all selected articles exclusively by their titles and abstract. The second step involved screening the remaining articles, analyzing their full text. We resolved discrepancies by discussion, and if it was collectively agreed that a study did not meet the criteria for inclusion, it was excluded.

### 2.4. Data Extraction

Data from the included studies were extracted independently by the two reviewers, including the authors, publication year, country, study design, sample size, scar types treated (burns, keloids, and postoperative scars), characteristics of PRP administration, and outcome measurements (based on the Vancouver Scar Scale, Patient Observer Scar Assessment Scale, Visual Analog Scale, REEDA Scale, or by ultrasound and photographs).

### 2.5. Outcome of Interest and Definition

Scar improvement, as measured by recognized scoring systems, specifically the Vancouver Scar Scale (VSS), the Observer Scar Assessment Scale (OSAS), and the Patient and Observer Scar Assessment Scale (POSAS), were the main objectives. VSS assesses vascularity, pigmentation, pliability, and height on a scale of 0 to 13, with higher scores suggesting worst scarring [13]. OSAS, a component of POSAS, judge scars based on six parameters: vascularity, pigmentation, thickness, relief, pliability, and surface area. Both VSS and OSAS are clinician determined. Patient Scar Assessment Scale (PSAS) is based on patient judgement and is the other component of POSAS, which is a dual-component scale that evaluates both patient-reported and observer-reported assessments, with a score ranging from 6 (best) to 60 (worst) [14]. A statistically significant decrease in the overall or subscale scores on these measures were considered scar improvement.

The timing of outcome measurement differed among the studies; however, the majority evaluated scar outcomes between one and six months following the final PRP treatment. Long-term effects (≥12 months) and follow-up intervals were also extracted when available.

### 2.6. Quality Assessment

The quality and risk of bias of the selected studies were evaluated using version 2 of the Cochrane Risk of Bias tool for randomized trials (RoB 2) through five key domains: (1) randomization process, (2) deviations from intended interventions, (3) missing outcome data, (4) measurements of the outcome, and (5) selection of the reported results. Depending on these five items, we ranked the research quality as either ‘high risk,’ ‘low risk,’ or “some concerns”.

### 2.7. Statistical Analysis

A random effect meta-analysis was performed using Comprehensive Meta-Analysis (CMA) software, version 3.3. Statistical heterogeneity across the studies was assessed using the Higgins I^2^ ≤ 25% for low heterogeneity, 25–50% for moderate heterogeneity, and ≥50% for high heterogeneity [15]. Since the number of studies was fewer than 10, a funnel plot analysis was not carried out. Additionally, an Egger’s test was conducted for three or more studies. Statistical significance was determined at *p* < 0.05.

## 3. Results

### 3.1. Literature Search

**Figure 2:**
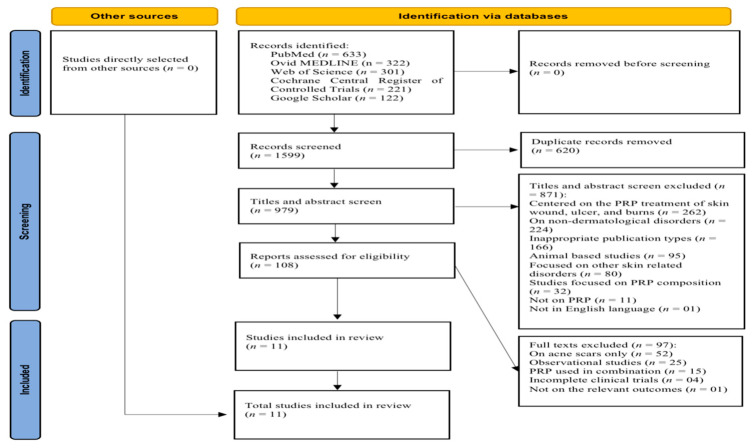
PRISMA 2020 flowchart of the searching and screening studies.

The search of five databases resulted in the identification of 1599 studies. After excluding 620 duplicates, 979 studies were considered for title and abstract evaluation. At this stage, 871 studies were excluded for different reasons, as described in the PRISMA flowchart in Figure 1. Of the 97 full-text studies that remained, 52 were focused on acne scars only, 25 were observational studies, 15 examined PRP in combination with other treatments, 4 were incomplete clinical trials, and 1 did not focus on the relevant outcomes. In the final analysis, 11 articles were selected (Figure 2).

### 3.2. Quality Assessment of Included Studies

Based on the updated RoB 2 tool’s assessment criteria [16], six RCTs in the systematic review were rated as having a low risk of bias. Four studies were found to have a high risk of bias, and one study was categorized as having some concerns. The main factor contributing to the high risk of bias was the domain that pertains to bias in the measurement of outcomes. The study with some concerns was attributed to noncompliance with the domain regarding deviations from the intended interventions. The quality assessment of the included studies is detailed in Table 1.

### 3.3. Baseline Characteristics of Included Studies

The included randomized controlled trials (RCTs) were conducted in several countries, with four studies from Egypt, three from Iran, and one each from Spain, Kazakhstan, The Netherlands, and Poland. Sample sizes varied significantly, ranging from 10 to 160 participants, and totaling 610 individuals. The participants’ mean age varied from 4.3 months to 51.2 years, with an overall mean age of 31 years, and 70.1% of participants were female.

The scar types were mainly postoperative scars, in five studies, followed by burn scars in four studies. Two studies were focused on keloids, and one was centered on cheiloplasty scars. To evaluate the scar quality, the outcomes of the Postoperative Scar Assessment Score (POSAS) were applied in six studies, the Visual Scoring System (VSS) in three, and scar dimensions (e.g., width or thickness) were measured in two studies. One remaining study utilized both the POSAS and VSS. Follow-up periods also varied, with most studies (*n* = 5) having a 3-month follow-up, while three studies followed participants for 6 months, two for 12 months, and one for 2 months. The baseline characteristics of the included studies are presented in Table 2.

### 3.4. PRP Preparation and Administration

PRP preparation and administration protocols across the included studies were highly variable but generally followed similar principles.

Blood Collection and Anticoagulation: Most studies used vacutainer kits with sodium citrate as an anticoagulant to prevent clotting during blood collection. Blood volumes varied, with some studies using 10 mL [23,24] and others using up to 30 mL [17,18]. One study also used specialized tubes with thixotropic gel [18] to facilitate the separation of blood components.

PRP Separation and Composition: The separation of PRP from other blood components involved different centrifugation techniques, i.e., both two-step centrifugation ([17,23]) and a single-spin method [18,21]. The PRP yield ranged from 4 mL to 10 mL per patient depending on the blood volume and centrifugation method.

Platelet Activation: Platelet activation was typically achieved by adding calcium chloride (CaCl2) [21] or thrombin [18]. In some protocols, PRP was subjected to freeze–thaw cycles to reduce inflammatory mediators [21].

PRP Administration: PRP was injected intradermally or at the site of injury. One study used syringes with atomizers for more precise distribution [21]. The dosage of PRP per injection ranged from 0.25 mL [24] to 1.5 mL per site [21] (Table 3).

### 3.5. Interventional Characteristics of Included Studies

To evaluate the interventional characteristics, the studies were categorized based on the type of scar.

#### 3.5.1. Postoperative Scars

Five studies assessed the impact of one session of PRP in comparison to control on postoperative scars, with three of these studies (*n* = 176) focusing on cesarean section surgeries. The follow-up period was three months, except for the studies of Tehranian et al., who followed the patients for 2 months [26], and Refahee et al., who followed up for 6 months [24]. In all these studies, a significant improvement in scar quality and appearance was observed with PRP treatment compared to the control group.

There were inconsistencies in the outcome assessments (POSAS, VSS, and scar thickness) across three studies involving cesarean section patients. The first study of Barwijuk et al. found significant differences in scar quality between the PRP and control groups, with both patient and observer assessments (using the POSAS scale) showing improved results for the PRP group at 30 and 90 days (*p* < 0.05) post-surgery. However, differences on day 8 (*p* > 0.05) were not statistically significant [18]. Similarly, Tehranian et al. reported a 93% reduction in the VAS score for PRP-treated patients by the end of the follow-up (2 months), compared to only a 79% reduction in the control group (*p* < 0.001) [26]. Chaichian et al. also observed that scar thickness at the 12th week after surgery was significantly reduced in the PRP group, with the scar being about one-fourth the size of that in the control group (*p* = 0.002) [19].

The one study (*n* = 100) focusing on maxillofacial surgery also demonstrated that PRP treatment resulted in significantly better aesthetic outcomes. Thirty days post-surgery, the treatment group had a mean POSAS score of 2.5, while the control group scored 5.8 (*p* < 0.05). At 90 days, the treatment group showed a mean score of 1.6, while the control group scored 3.7 (*p* < 0.05) [22].

The remaining RCT (*n* = 24) assessed the effect of PRP vs. control on cheiloplasty scars following unilateral complete cleft lip repair. The study involved one session of PRP, and scar width was measured using ultrasound after 6 months. The results showed a significant difference in scar width between the control and PRP groups. The mean scar width was 4.96 ± 0.929 mm in the control group and 3.8 ± 0.886 mm in the PRP group, with a statistically significant improvement (*p* = 0.0047) in the PRP-treated group [24].

#### 3.5.2. Burn Scars

There were four studies that evaluated the effect of PRP on burn scars, with follow-up periods ranging from three to twelve months. While PRP shows promise in enhancing burn scar healing, its effects remain inconsistent.

Among the four studies, two found significant improvements in scar quality and appearance with PRP treatment. Roohaninasab et al. (*n* = 10) showed that PRP significantly enhanced burn scar healing compared to placebo, as assessed by the VSS after three months (*p* = 0.001), with two sessions conducted at one-month intervals [17]. However, the combination of PRP with non-cross-linked hyaluronic acid (HA) showed even more pronounced results. Similarly, Elsayed et al. (*n* = 38) found that PRP led to improved scar appearance after six months, with PRP being safe and effective, though the comparison was made with silicone-based products using the POSAS score [25].

While García-Sánchez et al. (*n* = 20) observed lower VSS and POSAS scores for PRP compared to the controls at six months, the differences were not statistically significant [21]. Similarly, Marck et al. (*n* = 12) also found no significant differences in the PSOAS scores between PRP-treated and control areas at 12 months [27].

#### 3.5.3. Keloids

There were two studies that evaluated the impact of PRP on keloids, with follow-up periods of 3 [23] and 12 [20] months.

The first study involved 160 cases, divided into four groups, each containing 40 cases. Group A (control) received intralesional triamcinolone, Group B received intralesional verapamil, Group C received intralesional 5-fluorouracil, and Group D received intralesional PRP. The baseline POSAS scores were similar across all groups, ranging from 89 to 92. After 24 weeks, significant differences were observed between the groups, with PRP showing improvement similar to triamcinolone. However, intralesional verapamil was reported as the most effective treatment [20].

In the second study, 60 keloid patients were randomly assigned to three groups: one treated with botulinum toxin type-A (BTX-A), another with PRP, and the third with triamcinolone acetonide (TAC). Significant improvement in the VSS scores was observed in both the BTX-A and PRP groups compared to the TAC group (*p* < 0.001), though no significant difference was found between BTX-A and PRP (*p* = 0.422) [23] (Table 4).

### 3.6. Meta-Analysis

The meta-analysis evaluated six outcomes to assess the effects of PRP and control on scar appearance and quality, based on OSAS, PSAS, and VSS scores.

#### 3.6.1. Comparison of OSAS Scores at Three-Month Follow-Up

**Figure 3:**
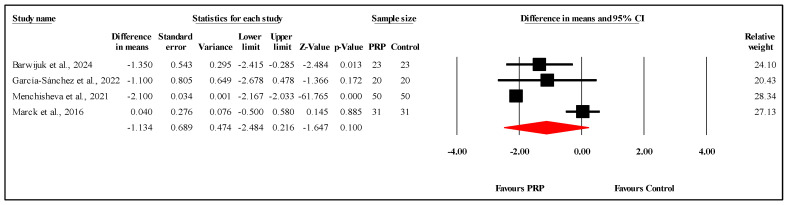
Forest plot comparison of OSAS scores between PRP and control groups at three-month follow-up [18,21,22,27]. Note: Each black square represents an individual study’s effect size, with the line showing its 95% confidence interval. The red diamond represents the overall pooled effect size, and its width indicates the 95% confidence interval for the combined result.

The meta-analysis of four studies [18,21,27] assessing OSAS scores between PRP and control groups at the three-month follow-up found no significant differences. The overall mean difference (MD) was −1.134, suggesting no statistically significant impact of PRP on scar outcomes at the three-month mark, with a 95% confidence interval (CI) of −2.484 to 0.216 and a nonsignificant result (*p* = 0.100) (Figure 3). The analysis revealed very high heterogeneity (I^2^ = 95.2%), reflecting substantial variability among the included studies. Furthermore, Egger’s test indicated significant publication bias, with an intercept of −19.18 (95% CI: −28.10 to −10.25) and a two-tailed *p*-value of 0.0115. Refer to Supplementary Materials Table S1 for heterogeneity and T2, Figure S1 for the funnel plot and Table S2 for Egger’s test results. 

#### 3.6.2. Comparison of OSAS Scores at Six-Month Follow-Up

**Figure 4:**
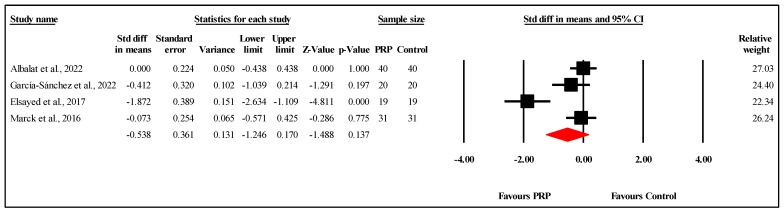
Forest plot comparison of OSAS scores between PRP and control groups at six-month follow-up [20,21,25,27].

The meta-analysis of four studies [20,21,25,27] comparing the OSAS scores between PRP and control groups at the six-month follow-up found no statistically significant difference. The overall MD was −0.591, with a 95% CI ranging from −1.190 to 0.008 and a marginally nonsignificant result (*p* = 0.053) (Figure 4). The heterogeneity among the studies was moderate (I^2^ = 55.7%, *p* = 0.080), indicating some variability in the results. Egger’s test for publication bias revealed a marginally significant bias, with an intercept of −9.86 (95% CI: −22.82 to 3.10) and a two-tailed *p*-value of 0.082, suggesting that publication bias may have modestly influenced the findings. Refer to Supplementary Materials Table S3 for heterogeneity and T2, Figure S2 for the funnel plot and Table S4 for Egger’s test results. 

#### 3.6.3. Comparison of PSAS Scores at Three-Month Follow-Up

**Figure 5:**
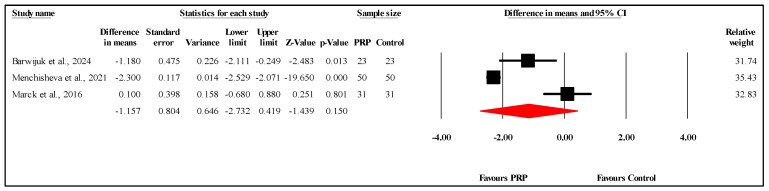
Forest plot comparison of PSAS scores between PRP and control groups at three-month follow-up [18,22,27].

The meta-analysis of three studies [18,27] comparing PSAS scores between PRP and control groups at the three-month follow-up showed no statistically significant difference. The overall MD was −1.157, with a 95% CI ranging from −2.732 to 0.419 and a nonsignificant result (*p* = 0.150) (Figure 5). The analysis demonstrated very high heterogeneity (I^2^ = 94.6%, *p* < 0.001), indicating substantial variability in the results. Egger’s test for publication bias revealed no significant bias, with an intercept of −40.57 (95% CI: −274.82 to 193.69) and a two-tailed *p*-value of 0.271, suggesting that publication bias is unlikely to have affected the findings. Refer to Supplementary Materials Table S5 for heterogeneity and T2, Figure S3 for the funnel plot and Table S6 for Egger’s test results.

#### 3.6.4. Comparison of PSAS Scores at Six-Month Follow-Up

**Figure 6:**
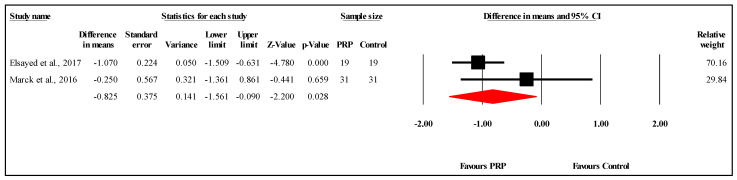
Forest plot comparison of PSAS scores between PRP and control Groups at six-month follow-up [25,27].

The meta-analysis of two studies comparing PSAS scores between PRP and control groups at the six-month follow-up demonstrated a statistically significant difference favoring PRP. The overall MD was −0.825, with a 95% CI of −1.561 to −0.090 and a significant result (*p* = 0.028) (Figure 6). The heterogeneity was low to moderate (I^2^ = 44.8%, *p* = 0.178), indicating limited variability between the study results. Refer to Supplementary Materials Table S7 for heterogeneity and T2. Funnel plot analysis or Egger’s test could not be conducted, as they require at least three studies.

#### 3.6.5. Comparison of VSS Scores at One-Month Follow-Up

**Figure 7:**
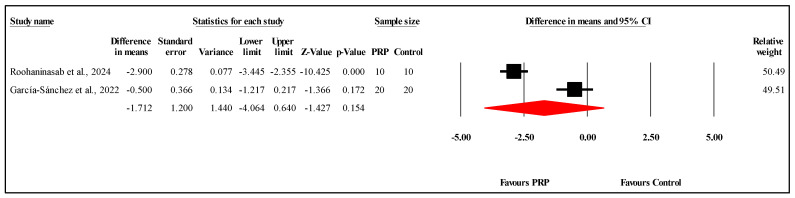
Forest plot comparison of VSS scores between PRP and control groups at one-month follow-up [17,21].

The meta-analysis of two studies [21] comparing VSS scores between PRP and control groups at the one-month follow-up showed no statistically significant difference. The overall MD was −1.712, with a 95% CI of −4.064 to 0.640 and a nonsignificant result (*p* = 0.154) (Figure 7). The heterogeneity among the studies was very high (I^2^ = 96.3%, *p* < 0.001), indicating substantial variability in the results. Refer to Supplementary Materials Table S8 for heterogeneity and T2. Funnel plot analysis or Egger’s test could not be conducted, as they require at least three studies.

#### 3.6.6. Comparison of VSS Scores at Three-Month Follow-Up

**Figure 8:**
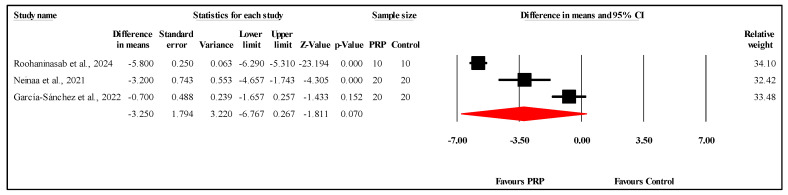
Forest plot comparison of VSS scores between PRP and control groups at three-month follow-up [17,21,23].

The meta-analysis of three studies comparing VSS scores between the PRP and control groups at the three-month follow-up showed no statistically significant difference. The overall MD was −3.250, with a 95% CI of −6.767 to 0.267 and a borderline nonsignificant result (*p* = 0.070) (Figure 8). The heterogeneity was very high (I^2^ = 97.8%, *p* < 0.001), indicating substantial variability among the study results. Egger’s test for publication bias revealed no significant bias, with an intercept of −7.11 (95% CI: −30.30 to 16.08) and a two-tailed *p*-value of 0.160. Refer to Supplementary Materials Table S9 for heterogeneity and T2, Figure S4 for the funnel plot and Table S10 for Egger’s test results.

## 4. Discussion

Scarring can have both aesthetic and functional consequences, often impairing quality of life [6]. Platelet-rich plasma (PRP), due to its high concentration of growth factors, cytokines, and bioactive molecules, has been used as a therapeutic option for improving scar quality [28]. This meta-analysis included 11 randomized controlled trials involving 539 patients in total, evaluating the efficacy of PRP in treating burns, keloid, and postoperative scars using validated scar assessment tools. Although some individual studies demonstrated positive outcomes, the overall data show no significant changes in scar appearance based on the OSAS and VSS scores at the 3-month follow-up. However, PRP showed a statistically significant improvement in PSAS scores at the 6-month follow-up, indicating possible improvement with chronic use. Discrepancies in results may be partially explained by differences in scoring systems, PRP preparations and administration, and scar types.

Scar improvement is commonly evaluated using standardized clinical tools, which, in this study, primarily focused on three main systems: the Patient Scar Assessment Scale (PSAS), the Observer Scar Assessment Scale (OSAS), and the Vancouver Scar Scale (VSS). The PSAS reflects the patient’s personal perception of their scar, placing particular emphasis on quality of life. It assesses six self-reported factors: pain, itchiness, color, stiffness, thickness, and irregularity. In contrast, the OSAS and VSS focus solely on clinician observations. The OSAS evaluates six parameters: vascularity, pigmentation, thickness, relief, pliability, and surface area. Both the PSAS and OSAS use a 10-point scale for each item, where 1 represents normal skin and 10 indicates the worst possible scar, resulting in total scores ranging from 6 to 60. Developed in 2004, the Patient and Observer Scar Assessment Scale (POSAS) combines both perspectives by including two components: OSAS (completed by the clinician) and PSAS (completed by the patient). The overall POSAS score is the sum of both assessments [14]. VSS was introduced in 1990, initially designed to evaluate burn scars, and recently has been used for post-surgical scars. It strictly consists of four clinician rated parameters: vascularity, pigmentation, pliability, and height, with scores ranging from 0 (normal skin) to 13 (worst scar tissue) [13].

This distinction between patient and clinician-based assessments may explain why studies using the PSAS reported statistically significant outcomes at six months, as it captures subjective symptoms, such as pain, itchiness, and stiffness, which are not fully captured by clinician-assessed scales [29]. While patient-reported outcomes, such as those measured by the PSAS, provide valuable insight into how patients perceive scar appearance and its impact on quality of life, they are fundamentally subjective and susceptible to bias. Systematic reviews have demonstrated that patient perception can be influenced by depression, placebo effects, inter-rater variability, and lack of reproducibility [30,31]. In contrast, objective tools, such instrumental assessments (ultrasound imaging, skin elasticity testing, or colorimetry) provide quantifiable and standardized evaluations [32]. A more balanced use of both subjective and objective measures in future studies would enhance the reliability and clinical relevance of PRP outcome data.

To improve clinical comparability, the PRP protocols used in the included studies could be classified using the PAW approach (platelet concentration, activation technique, and white blood cell content) or similarly the DEPA classification (dose of injected platelets, efficiency of production, purity of the PRP, and activation of the PRP) [33,34]. Although certain methodological data were available, many of the included studies did not provide complete reporting on crucial characteristics, such as exact platelet concentration, leukocyte content, PRP yield, and activation status (Table 3). As a result, the PAW and DEPA methods could not be used to classify the PRP treatments comprehensively and consistently. Nonetheless, a qualitative assessment revealed considerable variability in the PRP preparation protocols, which is likely due to the absence of a universally standardized protocol in the field [35].

Differences were observed in the volume of blood collected (10 mL or 30 mL), the type of anticoagulant used (sodium citrate vs. thixotropic gel), centrifuge method (single vs. double spin), platelet activation (optional), and the final concentration of PRP. These variations may have influenced treatment outcomes. Although protocols vary, they generally follow the same sequence: blood collection, centrifugation to isolate and concentrate platelets, extraction of PRP, activation with a platelet-stimulating agent (optional), and lastly, application to the treatment site [35]. This heterogeneity highlights a critical gap in the PRP literature and shows the need for standardized preparation and classification criteria in future trials to enable meaningful comparisons between studies and to optimize clinical recommendations.

One of these variations included centrifugation protocols, which affect the platelet yield and cellular composition differently. Bhatia et al. reported that the single-spin method may be more effective in concentrating platelets [36]. In contrast, Saqlain et al. found that the double-centrifugation method resulted in higher platelet counts and, therefore, is more beneficial for preparing autologous and allogenic PRP [37]. However, despite these methodological differences, clinical outcomes have consistently shown improvement in scar healing, regardless of the preparation technique used. Roohaninasab et al. used a double-spin method for PRP preparation in burn scar treatment. They reported a significant reduction in VSS scores over three months, from 9.3 (pre-treatment) to 3.2 (post-treatment); an overall 6.1-point improvement [17]. On the other hand, Barwijuk et al. employed a single-spin PRP method during cesarean section wound closure. The results showed statistically significant improvements in scar quality, as assessed by the POSAS at day 90: patient *p* = 0.021 and observer *p* = 0.002 [18].

Among the scar types included, postoperative scars demonstrated the most consistent and significant improvements with PRP treatment, as evidenced by all five relevant RCTs reporting superior outcomes compared to the controls. In contrast, burn scar studies produced mixed results, with only two showing statistically significant improvements. García-Sánchez et al. found that while VSS and POSAS scores were lower in the PRP group at six months, the differences were not statistically significant [21]. Similarly, Marck et al. observed no significant differences in POSAS scores between the PRP and control areas at 12 months [27]. Keloid-related studies showed that PRP showed improvement but was generally comparable to or less effective than conventional treatments, like triamcinolone or verapamil. These patterns suggest that PRP may be most beneficial in acute, surgically induced scars, while its role in chronic or fibrotic scar types, like keloids, remains less well defined.

While PRP has been evaluated in various scar types, most of our patients applied PRP during the early stages of wound healing, such as immediately post-surgery or within the first few weeks following injury or excision. This early application coincides with the inflammatory and proliferative phases of healing, when growth factors can exert maximal regenerative effects and may augment the body’s natural repair mechanisms by enhancing angiogenesis, fibroblast migration, and extracellular matrix production [22,38]. In contrast, applying PRP in chronic or fibrotic scars, which are characterized by elevated protease activity leading to continuous degradation of matrix proteins and growth factors, presents challenges [39]. The efficacy of PRP can be compromised by this process. Anitua et al. noted that wounds can achieve 80–100% epithelialization in as early as 4 days; however chronic wounds achieved 90–100% epithelialization within 15 days [38]. This underscores the significance of wound chronology in the planning of PRP treatment and implies that the optimal timing for PRP application is a critical area for future research.

Elmarakby et al. investigated platelet-rich plasma (PRP) in comparison to other biologically active treatments, such as nano fat grafting in stimulating tissue remodeling and scar improvement in recent post-traumatic scars. Both are autologous and minimally invasive, leveraging biologically active components: PRP through platelet-derived growth factors, and nano fat through adipose-derived stem cells (ADSCs). Although both treatments resulted in some level of improvement, nano fat demonstrated significantly superior outcomes, as assessed by the Vancouver Scar Scale (VSS). Specifically, the nano fat group improved from a baseline VSS of 8.10 ± 1.29 to 3.10 ± 2.18 at 3 months, and further to 2.50 ± 1.96 at 6 months. In contrast, the PRP group showed more modest improvements, with VSS scores decreasing from 8.40 ± 1.65 to 5.20 ± 1.32 at 3 months and to 4.80 ± 1.81 at 6 months [40].

Although PRP monotherapy yields promising improvements, its true potential may be realized when combined with established modalities. Ebrahimi et al. supports this by showing a moderate observed degree of response (25–50%), with PRP alone in 36% of the population. However, when combined, PRP + CO_2_ fractional laser, 43% of patients achieved marked improvement (50–75%) and 32% achieved excellent improvement (>75%), versus much lower rates with laser alone. Similarly, in trials of microneedling ± PRP, marked improvement was seen in 43% of PRP-treated sides versus around 25% with microneedling alone, and excellent outcomes occurred in 23% versus 8% without PRP [41]. While Ebrahimi et al. emphasized combination therapies, our study contributes to the field by providing a focused evaluation of PRP’s independent effectiveness over specific scar types, allowing for more tailored clinical insights by scar etiology.

In addition to scar management, PRP has been investigated in a variety of clinical settings, including alopecia, wound management (ulcers), musculoskeletal settings (knee osteoarthritis, lateral epicondylitis), and neurosurgical conditions (facilitating dural closure and treating carpel tunnel syndrome) [42,43,44,45,46]. The economic value of PRP appears to vary by clinical context. For example, research on chronic ulcers has demonstrated that PRP can be cost-effective, leading to enhanced healing outcomes and decreased long-term care expenses when contrasted with conventional therapies [43]. In contrast, PRP has been demonstrated to be more costly than the standard treatment for osteoarthritis, such as corticosteroid injections, but with more significance in improvement [47]. Notably, there are currently no economic evaluations assessing the cost-effectiveness of PRP specifically for scar management. Future research evaluating the cost-effectiveness of PRP treatment in comparison to other modalities should be incorporated into future research to better assist clinical decision-making in this area.

This study has several limitations that must be taken into consideration when interpreting the results. High heterogeneity was observed in some of the outcomes, including the OSAS and PSOS scores at the three-month follow up and VSS scores at both follow-up intervals. This is likely due to variability in PRP preparation methods, administration protocols, and scar types. In addition, Egger’s test indicated publication bias specifically in OSAS outcomes. However, despite our study including 11 papers, which exceeds the recommended minimum for such a test (10–20 studies), the limited sample size may still limit the test’s sensitivity [48]. Several included RCTs were rated as high risk of bias from measurements of the outcome, with most showing some concerns related to bias due to deviations from the intended interventions. These methodological limitations may have potentially inflated the treatment effects. Although a sensitivity analysis was not performed, these factors should be considered when interpreting the results. Some studies had small samples sizes compared to the others, and most follow-up assessments in the literature were conducted at relatively short intervals, typically at 1, 3, and 6 months. Only a few studies, such as that by Marck et al. [27], extended follow-up to 12 months. Similarly, most studies assessed outcomes using subjective scar scales, with limited incorporation of imaging-based assessments, as carried out in Refahee et al.’s study using ultrasound [24].

Future research should incorporate extended follow-up periods beyond 12 months to comprehensively evaluate sustained efficacy, better understand PRP’s role in long-term scar remodeling, refine treatment protocols accordingly, assess the cost effectiveness of PRP in scar management, and incorporate objective assessment tools.

## 5. Conclusions

The aim of our systemic review and meta-analysis was to review the possible benefits of PRP in improving scar quality. According to our results, PRP showed statistically significant improvement at the 6-month follow-up according to the PSAS score, which emphasizes PRP’s role in relieving subjective symptoms, such as pain, itchiness, and stiffness. These are factors that directly influence quality of life but may be unnoticed in clinician-based assessments. This conclusion is derived from a restricted quantity of studies and must be regarded with caution. Despite most individual studies showing improvement at early intervals, the pooled analysis resulted in no significant changes in scar appearance at a 3-month interval. The variability in the results, particularly in burn and keloid scars, indicates that PRP may not be universally effective. Future studies should examine the use of PRP for fibrotic or chronic scars, standardize PRP procedures, and examine long-term results. PRP is still a promising adjunct until then, particularly in the early stages of wound healing, when it may have its greatest regenerative effects.

## Figures and Tables

**Table 1:** Assessment of quality of included studies using 2019 updated Cochrane Risk of Bias tool (RoB 2) [16].

Criterion	[17]	[18]	[19]	[20]	[21]	[22]	[23]	[24]	[25]	[26]	[27]
D1	🟢	🟢	🟢	🟢	🟢	🟢	🟢	🟢	🟢	🟢	🟢
D2	🟢	🟨	🟢	🟨	🟢	🟨	🟨	🟢	🟨	🟢	🟢
D3	🟢	🟢	🟢	🟢	🟢	🟢	🟢	🟢	🟢	🟢	🟢
D4	🟢	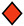	🟢	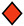	🟢	🟨	🟢	🟢	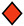	🟢	🟢
D5	🟢	🟢	🟢	🟢	🟢	🟢	🟢	🟢	🟢	🟢	🟢
Overall Risk	🟢	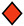	🟢	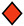	🟢	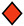	🟨	🟢	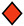	🟢	🟢

Note: 🟢 = low; 🟨 = some concerns; 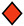 = high. D1: bias arising from the randomization process; D2: bias due to deviations from intended interventions; D3: bias due to missing outcome data; D4: bias in measurement of the outcome; D5: bias in selection of the reported result; D: overall risk of bias.

**Table 2:** Methodological characteristics of included studies.

Study	Design	Country	Sample Size and Demographics	Scar Type(s) Treated	PRP Administration	Outcome Measures
[17]	Double-blind RCT	Iran	*n* = 10; mean age: 42.2 (8.10) years; 80% female	Burn scars	Administered as 0.1 cc per point, with two sessions conducted at a 1-month interval.	VSS assessment before, 30, and 90 days after the first session
[18]	Single-blind placebo-controlled intervention study	Poland	*n* = 46; mean age: 31.1 (5.2) years; 100% female	Postoperative (CS) scars	A single session of PRP through a series of microinjections targeting the abdominal muscle fascia and the subcutaneous tissue.	POSAS assessment on days 1, 8, 30, and 90 after surgery
[19]	Randomized, double-blind pilot study	Iran	*n* = 30; mean age: 27.79 (6.8) years; 100% female	Postoperative (CS) scars	A single session of PRP delivered to both the upper and lower sides of the hysterotomy, at the junction between the decidua and myometrium.	Scar thickness (height) at 12th week after surgery
[20]	RCT	Egypt	*n* = 160; mean age: 32.3 (11.3) years; 68.1% female	Keloids	NR	POSAS assessment at baseline and 24th week
[21]	Single-center blinded RCT	Spain	*n* = 20; mean age: 40.05 (12.64) years; 25% female	Burn scars	A single PRP session, in which sterile syringes with an atomizer were used to administer 0.5 mL activator and 1.5 mL of PRP.	VS and POSAS assessment at baseline and at 1, 3, and 6 months
[22]	RCT	Kazakhstan	*n* = 100; mean age: 42 (5.5) years; 51% female	Postoperative facial scars	One PRP session (0.1–0.2 mL) were administered, with a spacing of 1.5–2 cm between each injection.	POSAS assessment on days 30 and 90 after surgery
[23]	Single-blind, randomized, comparative study	Egypt	*n* = 60; mean age: 25 (3.9) years; 40% female	Keloids	Three PRP sessions at 4-week intervals, with a 60° angle around the periphery of the keloids.	VSS assessment before, and 12th week after the first session
[24]	Randomized controlled clinical study	Egypt	*n* = 24; mean age: 4.3 (0.78) months; 37.5% female	Cheiloplasty scars	One session of PRP (0.25 mL) after muscle closure, injected on each side along the muscle and dermal suture lines.	Scar width (ultrasound) after 6 months
[25]	Prospective randomized clinical controlled trial	Egypt	*n* = 38; mean age: 19.8 (9.7) years; 63.2% female	Burn scars	Three PRP sessions, each administered monthly	POSAS assessment after 1 week, and 1, 3, and 6 months
[26]	RCT	Iran	*n* = 130; mean age: 28.8 (5.4) years; 100% female	Postoperative (CS) scars	One session of PRP was administered at the wound site, following fascia closure and before skin closure.	VSS assessment on day 1, day 8, and 8th week
[27]	Randomized, double-blind, intra patient-controlled study	The Netherlands	*n* = 52; mean age: 51.2 (18.6) years; 40% female	Burn scars	One PRP session with a two-syringe mechanism to ensure a 10:1 ratio of PRP and autologous clotting factors.	POSAS assessment at 3, 6, and 12 months

**Table 3:** Summary of variability in PRP protocol.

Study	Blood Collection	Anticoagulant	Centrifugation Method	Centrifugation Speed	Centrifugation Time	Activation	Total Injection Volume
[17]	30 mL	Sodium citrate (4%)	Double spin	500 rpm	8 min	Not specified	3 mL
[18]	24–30 mL	Thixotropic gel (2 mL)	Single spin	1500× *g*	5 min	Thrombin	8–10 mL
[19]	8.5 mL	Type not specified (1.5 mL)	Double spin	First: 1600 rpm Second: 3500 rpm	First: 10 min Second: 6 min	Not specified	2–3 mL
[20]	Not specified	Sodium citrate (2.4 mL)	Double spin	First: 2000 rpm Second: 5000 rpm	First: 3 min Second: 5 min	Calcium chloride (3%)	Not specified
[21]	54 mL	Acid citrate dextrose solution B	Single spin	400× *g*	7 min	Calcium chloride (10%)	1.5 mL PRP + 0.5 mL activator per treatment
[22]	9–27 mL (depending on wound)	Not specified	Single spin	3000 rpm	5 min	Not specified	0.1–0.2 mL
[23]	10 mL	Sodium citrate at 1:10	Double spin	First: 200× *g* Second: 1550× *g*	First: 10 min Second: 10 min	Not specified	0.1 mL per injection
[24]	10 mL	Sodium citrate (3.2%)	Double spin	First: 250× *g* Second: 1000× *g*	First: 10 min Second: 10 min	Not specified	1 mL
[25]	Not specified	Sodium citrate	Double spin	First: 400× *g* Second: 800× *g*	First: 10 min Second: 10 min	0.1 mL calcium gluconate per 0.9 mL PRP	0.2–0.3mL
[26]	55 mL	Citrate	Single spin	3200 rpm	15 min	Sodium bicarbonate	Not specified
[27]	54 mL	Citrate (6 mL)	Single spin	3200 rpm	15 min	Thrombin 10:1 ratio	Not specified

**Table 4:** Interventional characteristics of included studies.

Study	Intervention	Sample Size	OSAS Score	PSAS Score	VSS Score	Scar Thickness	Follow-Up	Conclusions
[17]	RPP	10			3.2 (0.42)	1826.1 (371.69)	3 months	PRP, non-cross-linked hyaluronic acid, and their combination are highly effective for burn scars.
	Control	10			9.0 (0.67)	1301.5 (189.89)		
	Non-cross-linked hyaluronic acid	10			5.7 (1.06)	1217.7 (297.3)		
	PRP-non-cross-linked hyaluronic acid	10			2.1 (0.99)	964.4 (285.91)		
[18]	PRP	23	13.39 (1.53)	14.91 (1.54)		2.65 (0.71)	3 months	PRP application notably enhanced scar healing in both the short and long term.
	Control	23	14.74 (2.11)	16.09 (1.68)		3.04 (0.64)		
[19]	PRP	15				2.1 (0.5)	3 months	Local PRP injection is an effective and practical approach to reducing scar thickness.
	Control	15				5.5 (0.8)		
[20]	Intralesional PRP	40	36 (12.74)				6 months	Intralesional verapamil is the most effective treatment, with PRP showing similar effectiveness to intralesional TAC.
	Intralesional triamcinolone	40	36 (12.74)					
	Intralesional verapamil	40	29 (10.91)					
	Intralesional 5-fluorouracil	40	38 (13.74)					
[21]	PRP	20	12		3.4	12	6 months	No statistical differences were found among the included groups.
	Control	20	13		4.4	13		
	PGRF	20	14		3.8	13		
[22]	PRP	50	1.6 (0.07)	2.7 (0.35)		1.7 (0.18)	3 months	PRP demonstrated a marked therapeutic effect on improving the esthetic outcomes of surgeries.
	Control	50	3.7 (0.23)	5.0 (0.75)		4.6 (0.15)		
[23]	Intralesional PRP	20			1.8 (2.3)		3 months	BTX-A and PRP may provide improved aesthetic results in keloid treatment over traditional TAC injections.
	Intralesional BTX-A	20			2.1 (2.6)			
	Intralesional TAC	20			5.0 (2.4)			
[24]	PRP	12				3.8 (0.886)	6 months	PRP treatment led to a significant reduction in scar width.
	Control	12				4.96 (0.929)		
[25]	PRP	19	3.36 (0.6)	3.48 (0.40)			6 months	PRP was found to be both safe and effective for treating scars.
	Silicone-based products	19	4.26 (0.32)	4.55 (0.89)				
[26]	PRP	67				1.11 (0.84)	2 months	PRP showed a significant reduction in keloid and hypertrophic scar formation.
	Control	71				1.7 (0.74)		
[27]	PRP	31	2.54 (0.91)	2.76 (1.44)		1.92 (0.99)	12 months	PRP did not result in enhanced scar quality.
	Control	31	2.55 (0.91)	3.05 (1.50)		2.16 (1.21)		

## Data Availability

All data supporting the findings of this study are contained within the article and its references.

## Supplementary Materials

The following supporting information can be downloaded at: https://www.mdpi.com/article/10.3390/jcm14238490/s1, Figures S1–S4: Funnel plot of observed and imputed studies; Tables S1, S3, S5, S7–S9: Heterogeneity and T2; Tables S2, S4, S6 and S10: The result of Egger’s test.

## Abbreviations

The following abbreviations are used in this manuscript:

PRP	Platelet-Rich Plasma
RCT	Randomized Controlled Trial
VSS	Vancouver Scar Scale
OSAS	Observer Scar Assessment Scale
PSAS	Patient Scar Assessment Scale
PDGF	Platelet-Derived Growth Factor
VEGF	Vascular Endothelial Growth Factor
EGF	Epidermal Growth Factor
FGF	Fibroblast Growth Factor
IGF-1	Insulin-Like Growth Factor
TGF-	Transforming Growth Factor-Beta
βHGF	Hepatocyte Growth Factor
KGF	Keratinocyte Growth Factor
BTX-A	Botulinum Toxin A
TAC	Triamcinolone Acetonide
CS	Cesarean Section
NR	Not Reported
