# Supplementary material for: The Efficiency of Platelet-Rich Plasma (PRP) in Treating Post-Burn and Surgical Scars: A Meta-Analysis Study
Source: J Clin Med. 2025 Nov 30;14(23):8490. doi: 10.3390/jcm14238490 (PMC12692934; doi:10.3390/jcm14238490)
Supplement: Supplementary file 1 [file jcm-14-08490-s001.zip › jcm-3748589-supplementary.pdf]

The analysis of Table *S*1 (Comparison of OSAS Scores at Three-Month Follow-Up)

Table S1 Heterogeneity and T2

Model	Effect size and 95% confidence interval						Test of null (2-Tail)		Heterogeneity				Tau-squared			
Model	Number Studies	Point estimate	Standard error	Variance	Lower limit	Upper limit	Z-value	P-value	Q-value	df (Q)	P-value	I-squared	Tau Squared	Standard Error	Variance	Tau
Fixed	4	-2.063	0.034	0.001	-2.129	-1.996	-60.588	0.000	62.537	3	0.000	95.203	1.673	1.992	3.968	1.293
Random	4	-1.134	0.689	0.474	-2.484	0.216	-1.647	0.100								

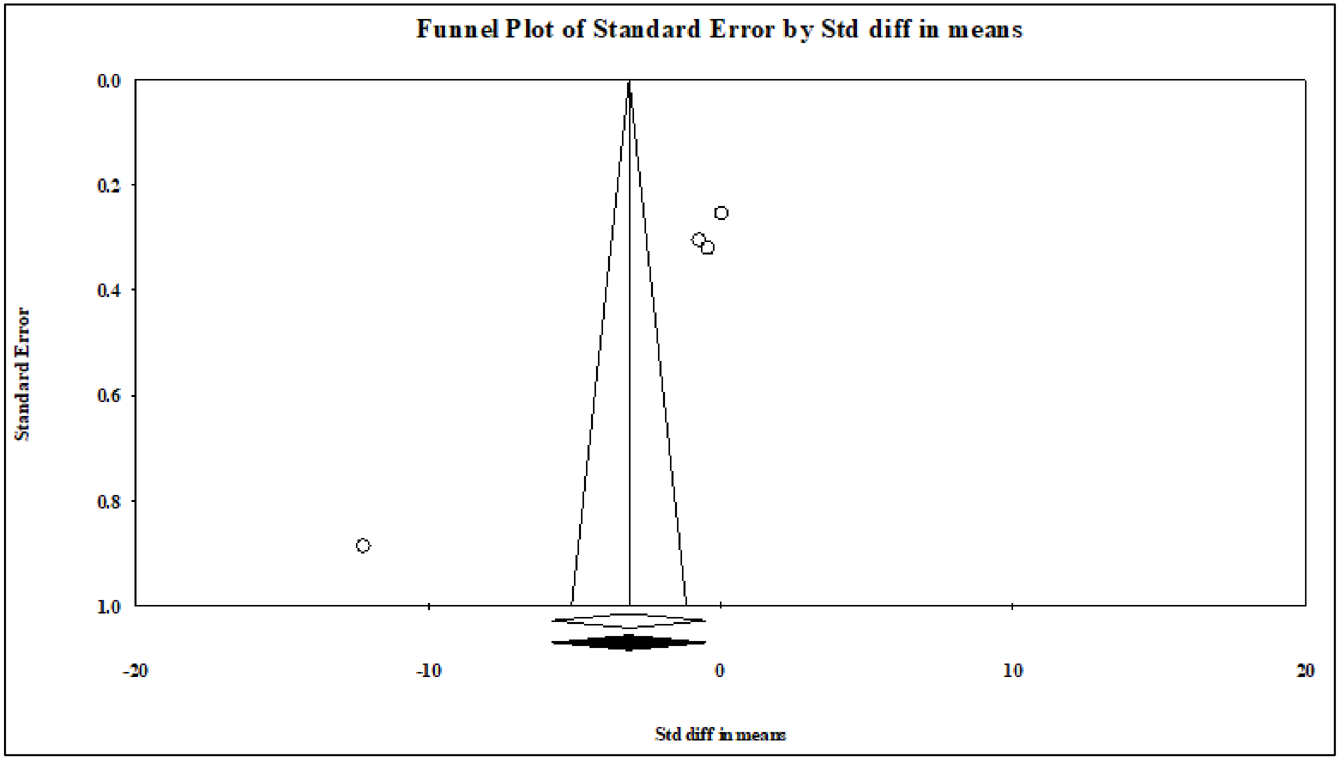

Figure S1 Funnel plot of observed and imputed studies

Table S2 The result of Egger's test

Egger's regression intercept

Intercept	-19.17857
Standard error	2.07427
95% lower limit (2-tailed)	-28.10345
95% upper limit (2-tailed)	-10.25369
t-value	9.24592
df	2.00000
P-value (1-tailed)	0.00575
P-value (2-tailed)	0.01150

The analysis of Table S**2 (**Comparison of OSAS Scores at Six-Month Follow-Up)

Table S3 Heterogeneity and T2

Model		Effect size and 95% confidence interval					Test of null (2-Tail)		Heterogeneity				Tau-squared			
Model	Number Studies	Point estimate	Standard error	Variance	Lower limit	Upper limit	Z-value	P-value	Q-value	df (Q)	P-value	I-squared	Tau Squared	Standard Error	Variance	Tau
Fixed	4	-0.338	0.139	0.019	-0.610	-0.066	-2.434	0.015	18.971	3	0.000	84.186	0.433	0.437	0.191	0.658
Random	4	-0.538	0.361	0.131	-1.246	0.170	-1.488	0.137								

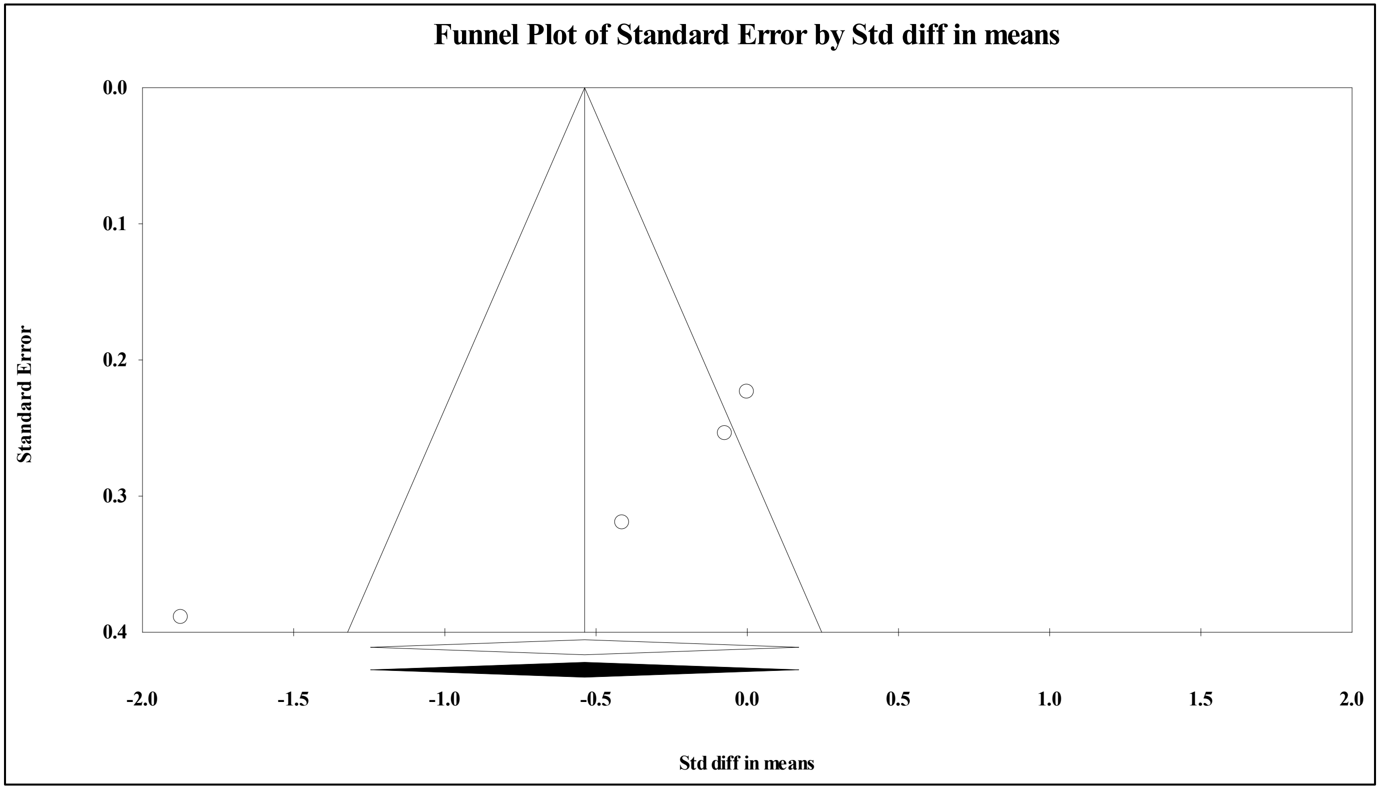

Figure S2 Funnel plot of observed and imputed studies

Table S4 The result of Egger's test

Egger's regression intercept

Intercept	-9.85796
Standard error	3.01269
95% lower limit (2-tailed)	-22.82050
95% upper limit (2-tailed)	3.10458
t-value	3.27215
df	2.00000
P-value (1-tailed)	0.04103
P-value (2-tailed)	0.08206

The analysis of Table S**3 (** Comparison of PSAS Scores at Three-Month Follow-Up)

Table S5 Heterogeneity and T2

Model		Effect size and 95% confidence interval					Test of null (2-Tail)		Heterogeneity				Tau-squared			
Model	Number Studies	Point estimate	Standard error	Variance	Lower limit	Upper limit	Z-value	P-value	Q-value	df (Q)	P-value	I-squared	Tau Squared	Standard Error	Variance	Tau
Fixed	3	-2.060	0.109	0.012	-2.274	-1.845	-18.848	0.000	37.124	2	0.000	94.613	1.810	2.095	4.389	1.345
Random	3	-1.157	0.804	0.646	-2.732	0.419	-1.439	0.150								

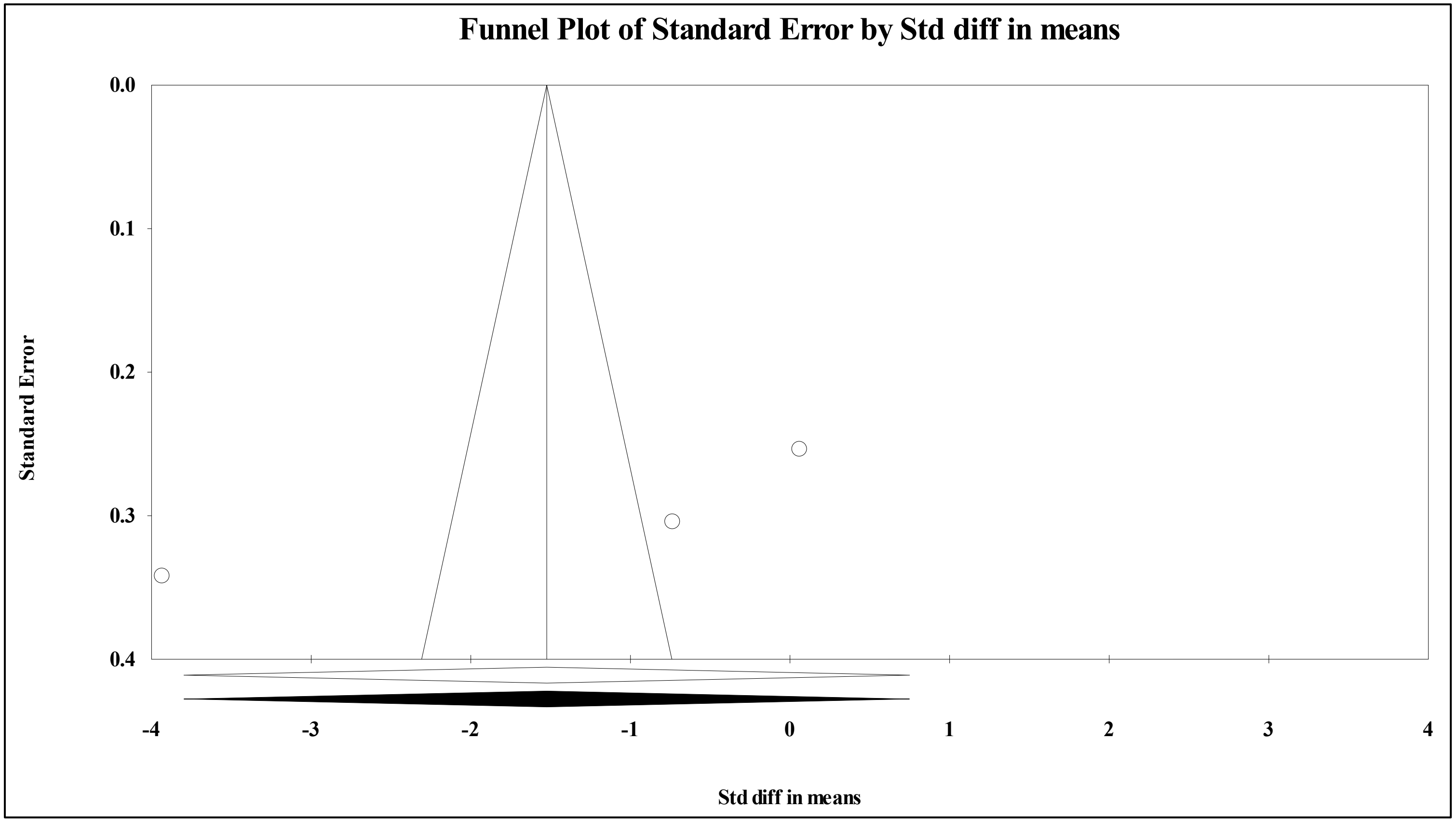

Figure S3 Funnel plot of observed and imputed studies

Table S6 The result of Egger's test

Egger's regression intercept

Intercept	-40.56613
Standard error	18.43618
95% lower limit (2-tailed)	-274.82003
95% upper limit (2-tailed)	193.68776
t-value	2.20035
df	1.00000
P-value (1-tailed)	0.13578
P-value (2-tailed)	0.27156

The analysis of Table *S*4 (Comparison of PSAS Scores at Six-Month Follow-Up)

Table S7 Heterogeneity and T2

Model		Effect size and 95% confidence interval					Test of null (2-Tail)		Heterogeneity				Tau-squared			
Model	Number Studies	Point estimate	Standard error	Variance	Lower limit	Upper limit	Z-value	P-value	Q-value	df (Q)	P-value	I-squared	Tau Squared	Standard Error	Variance	Tau
Fixed	2	-0.959	0.208	0.043	-1.367	-0.551	-4.608	0.000	1.811	1	0.178	44.772	0.151	0.475	0.226	0.388
Random	2	-0.825	0.375	0.141	-1.561	-0.090	-2.200	0.028								

The analysis of Table *S*5 (Comparison of VSS Scores at One-Month Follow-Up)

Table S8 Heterogeneity and T2

Model		Effect size and 95% confidence interval					Test of null (2-Tail)		Heterogeneity				Tau-squared			
Model	Number Studies	Point estimate	Standard error	Variance	Lower limit	Upper limit	Z-value	P-value	Q-value	df (Q)	P-value	I-squared	Tau Squared	Standard Error	Variance	Tau
Fixed	2	-2.021	0.221	0.049	-2.456	-1.587	-9.127	0.000	27.250	1	0.000	96.330	2.774	4.073	16.589	1.666
Random	2	-1.712	1.200	1.440	-4.064	0.640	-1.427	0.154								

The analysis of Table S6 **(**Comparison of VSS Scores at Three-Month Follow-Up**)**

Table S9 Heterogeneity and T2

Model	Effect size and 95% confidence interval						Test of null (2-Tail)		Heterogeneity				Tau-squared			
Model	Number Studies	Point estimate	Standard error	Variance	Lower limit	Upper limit	Z-value	P-value	Q-value	df (Q)	P-value	I-squared	Tau Squared	Standard Error	Variance	Tau
Fixed	3	-4.614	0.213	0.045	-5.032	-4.196	-21.639	0.000	90.325	2	0.000	97.786	9.379	10.691	114.299	3.062
Random	3	-3.250	1.794	3.220	-6.767	0.267	-1.811	0.070								

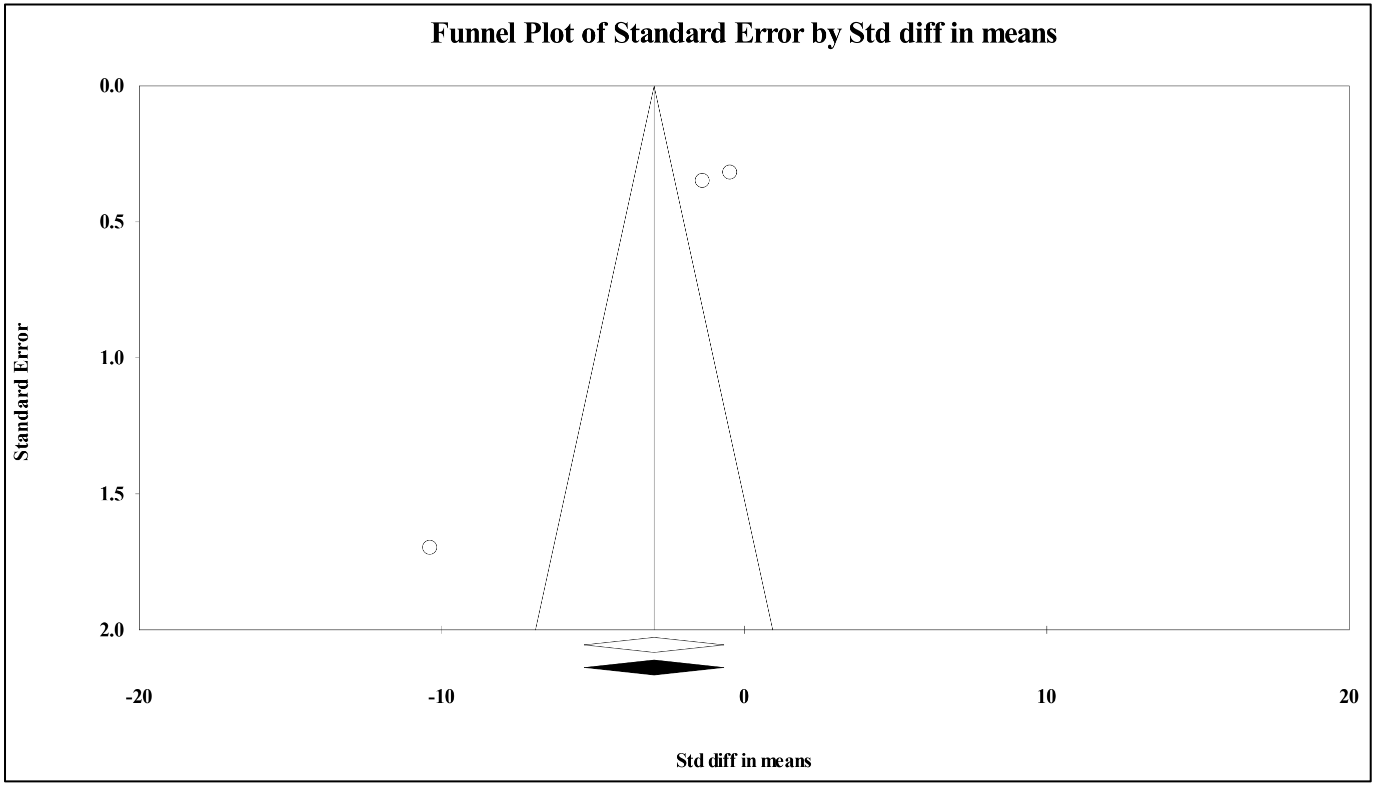

Figure S4 Funnel plot of observed and imputed studies

Table S10 The result of Egger's test

Egger's regression intercept

Intercept	-7.10909
Standard error	1.82512
95% lower limit (2-tailed)	-30.29942
95% upper limit (2-tailed)	16.08124
t-value	3.89514
df	1.00000
P-value (1-tailed)	0.07999
P-value (2-tailed)	0.15998
